# Supplementary material for: Reduction of Paraoxonase Expression Followed by Inactivation across Independent Semiaquatic Mammals Suggests Stepwise Path to Pseudogenization
Source: Mol Biol Evol. 2023 May 5;40(5):msad104. doi: 10.1093/molbev/msad104 (PMC10202596; doi:10.1093/molbev/msad104)
Supplement: msad104_Supplementary_Data [file msad104_supplementary_data.zip › SuppFig2_MinkBoxplotEnzymeActivities_022023_mod.pdf]

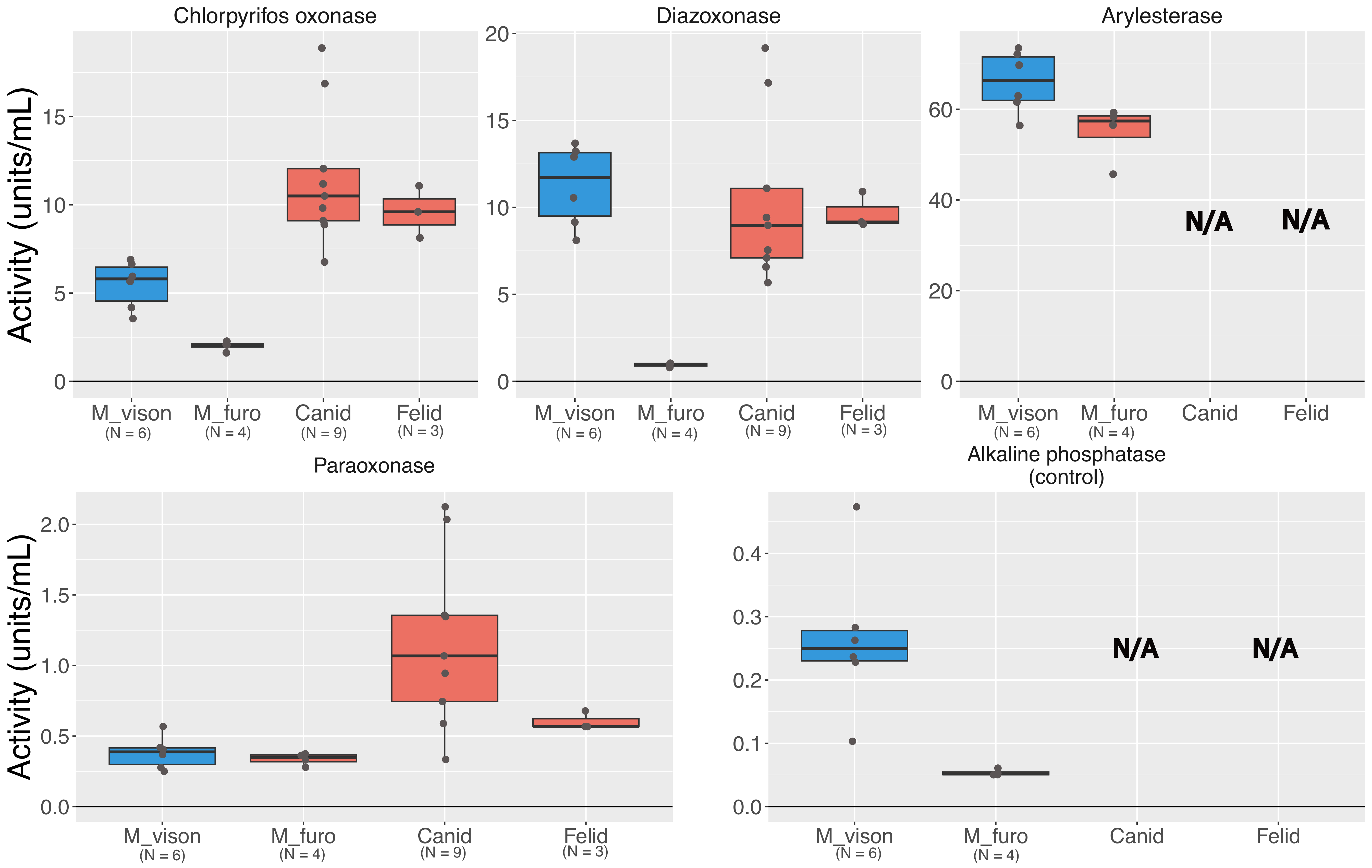

Chlorpyrifos oxonase
Diazoxonase
Arylesterase
Activity (units/mL)
0
5
10
15
20
40
60
M_vison
(N = 6)
M_furo
(N = 4)
Canid
(N = 9)
Felid
(N = 3)
N/A
N/A
Paraoxonase
Alkaline phosphatase
(control)
0.0
0.5
1.0
1.5
2.0
0.1
0.2
0.3
0.4
