# Supplementary material for: Reduction of Paraoxonase Expression Followed by Inactivation across Independent Semiaquatic Mammals Suggests Stepwise Path to Pseudogenization
Source: Mol Biol Evol. 2023 May 5;40(5):msad104. doi: 10.1093/molbev/msad104 (PMC10202596; doi:10.1093/molbev/msad104)
Supplement: msad104_Supplementary_Data [file msad104_supplementary_data.zip › Citations_from_supplemental.docx]

Acquarone M, Born EW, Speakman JR (2006). Field metabolic rates of walrus (Odobenus rosmarus) measured by the doubly labeled water method. *Aquat Mamm* **32**(3)**:** 363.

Ashwell-Erickson SM (1981). *The energy cost of free existence for Bering Sea harbor and spotted seals*. University of Alaska Fairbanks.

Ben-David M, Elias M, Filippi J-J, Duñach E, Silman I, Sussman JL *et al* (2012). Catalytic versatility and backups in enzyme active sites: the case of serum paraoxonase 1. *J Mol Biol* **418**(3-4)**:** 181-196.

Boveng PL, Bengtson JL, Buckley TW, Cameron MF, Dahle SP, Kelly BP *et al* (2009). Status review of the spotted seal (Phoca largha).

Boyd I, Duck C (1991). Mass changes and metabolism in territorial male Antarctic fur seals (Arctocephalus gazella). *Physiol Zool* **64**(1)**:** 375-392.

Briggs KT (1974). Dentition of the northern elephant seal. *J Mammal* **55**(1)**:** 158-171.

Butler PJ (2006). Aerobic dive limit. What is it and is it always used appropriately? *Comp Biochem Physiol, Part A Mol Integr Physiol* **145**(1)**:** 1-6.

Castellini MA, Kooyman GL, Ponganis PJ (1992). Metabolic rates of freely diving Weddell seals: correlations with oxygen stores, swim velocity and diving duration. *Journal of Experimental Biology* **165**(1)**:** 181-194.

Elgar M, Harvey PH (1987). Basal metabolic rates in mammals: allometry, phylogeny and ecology. *Funct Ecol***:** 25-36.

Ferren HJ (1980). Diving physiology of the ringed seal: adaptations, capability and implications. University of Alaska, Fairbanks.

Gerlinsky CD, Rosen DA, Trites AW (2013). High diving metabolism results in a short aerobic dive limit for Steller sea lions (Eumetopias jubatus). *Journal of Comparative Physiology B* **183:** 699-708.

Gjertz I, Griffiths D, Krafft B, Lydersen C, Wiig Ø (2001). Diving and haul-out patterns of walruses Odobenus rosmarus on Svalbard. *Polar Biol* **24:** 314-319.

Harel M, Aharoni A, Gaidukov L, Brumshtein B, Khersonsky O, Meged R *et al* (2004). Structure and evolution of the serum paraoxonase family of detoxifying and anti-atherosclerotic enzymes. *Nat Struct Mol Biol* **11**(5)**:** 412-419.

Hassrick J, Crocker D, Teutschel N, McDonald B, Robinson P, Simmons S *et al* (2010). Condition and mass impact oxygen stores and dive duration in adult female northern elephant seals. *Journal of Experimental Biology* **213**(4)**:** 585-592.

Hurley JA, Costa DP (2001). Standard metabolic rate at the surface and during trained submersions in adult California sea lions (Zalophus californianus). *Journal of Experimental Biology* **204**(19)**:** 3273-3281.

Josse D, Lockridge O, Xie W, Bartels CF, Schopfer LM, Masson P (2001). The active site of human paraoxonase (PON1). *Journal of Applied Toxicology: An International Journal* **21**(S1)**:** S7-S11.

Lenfant C, Johansen K, Torrance JD (1970). Gas transport and oxygen storage capacity in some pinnipeds and the sea otter. *Respir Physiol* **9**(2)**:** 277-286.

Lydersen C, Hammill M, Ryg M (1992). Water flux and mass gain during lactation in free‐living ringed seal (Phoca hispida) pups. *Journal of Zoology* **228**(3)**:** 361-369.

Peterson MW, Fairchild SZ, Otto TC, Mohtashemi M, Cerasoli DM, Chang WE (2011). VX hydrolysis by human serum paraoxonase 1: a comparison of experimental and computational results. *PLoS One* **6**(5)**:** e20335.

Plötz J, Bornemann H, Knust R, Schröder A, Bester M. (2002). *Ecological Studies in the Antarctic Sea Ice Zone: Results of EASIZ Midterm Symposium*. Springer, pp 148-156.

Ryg M, Are Øritsland N (1991). Estimates of energy expenditure and energy consumption of ringed seals (Phoca hispida) throughout the year. *Polar Res* **10**(2)**:** 595-602.

Scheffer VB, Slipp JW (1944). The harbor seal in Washington State. *The American Midland Naturalist* **32**(2)**:** 373-416.

Schreer JF, Kovacs KM, O'Hara Hines R (2001). Comparative diving patterns of pinnipeds and seabirds. *Ecol Monogr* **71**(1)**:** 137-162.

Shero MR, Andrews RD, Lestyk KC, Burns JM (2012). Development of the aerobic dive limit and muscular efficiency in northern fur seals (Callorhinus ursinus). *Journal of Comparative Physiology B* **182:** 425-436.

Slip DJ, Hindell MA, Burton HR (1994). Diving behavior of southern elephant seals from Macquarie Island: an overview. *Elephant seals: population ecology, behavior, and physiology University of California Press, Berkeley***:** 253-270.

Sterling J, Ream RR (2004). At-sea behavior of juvenile male northern fur seals (Callorhinus ursinus). *Can J Zool* **82**(10)**:** 1621-1637.

Stewart BS, DeLong RL (1995). Double migrations of the northern elephant seal, Mirounga angustirostris. *J Mammal* **76**(1)**:** 196-205.

Teilmann J, Born EW, Acquarone M (2000). Behaviour of ringed seals tagged with satellite transmitters in the North Water polynya during fast-ice formation. *Can J Zool* **77**(12)**:** 1934-1946.

Trites A, Bigg M (1996). Physical growth of northern fur seals (Callorhinus ursinus): seasonal fluctuations and migratory influences. *Journal of Zoology* **238**(3)**:** 459-482.

Weingartner GM, Thornton SJ, Andrews RD, Enstipp MR, Barts AD, Hochachka PW (2012). The effects of experimentally induced hyperthyroidism on the diving physiology of harbor seals (Phoca vitulina). *Frontiers in Physiology* **3:** 380.

Weise MJ, Costa DP (2007). Total body oxygen stores and physiological diving capacity of California sea lions as a function of sex and age. *Journal of Experimental Biology* **210**(2)**:** 278-289.

Williams TM, Haun J, Davis R, Fuiman L, Kohin S (2001). A killer appetite: metabolic consequences of carnivory in marine mammals. *Comp Biochem Physiol, Part A Mol Integr Physiol* **129**(4)**:** 785-796.

Yeung DT, Josse D, Nicholson JD, Khanal A, McAndrew CW, Bahnson BJ *et al* (2004). Structure/function analyses of human serum paraoxonase (HuPON1) mutants designed from a DFPase-like homology model. *Biochimica et Biophysica Acta (BBA)-Proteins and Proteomics* **1702**(1)**:** 67-77.

Yeung DT, Lenz DE, Cerasoli DM (2005). Analysis of active‐site amino‐acid residues of human serum paraoxonase using competitive substrates. *The FEBS journal* **272**(9)**:** 2225-2230.
