# Supplementary figures and images for: Reduction of Paraoxonase Expression Followed by Inactivation across Independent Semiaquatic Mammals Suggests Stepwise Path to Pseudogenization
Source: Mol Biol Evol. 2023 May 5;40(5):msad104. doi: 10.1093/molbev/msad104 (PMC10202596; doi:10.1093/molbev/msad104)

Paraoxonase

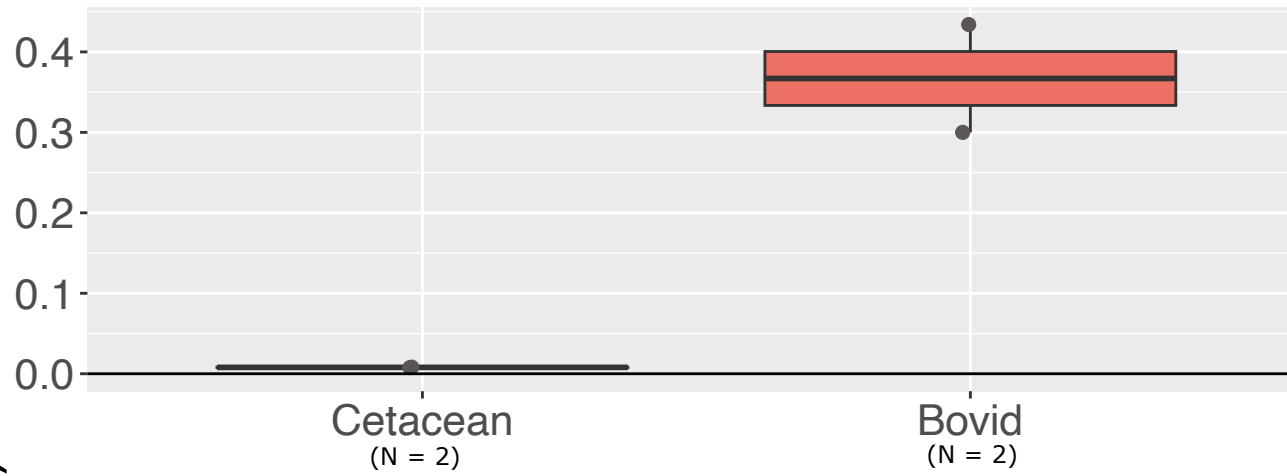

Alkaline phosphatase  
(control)

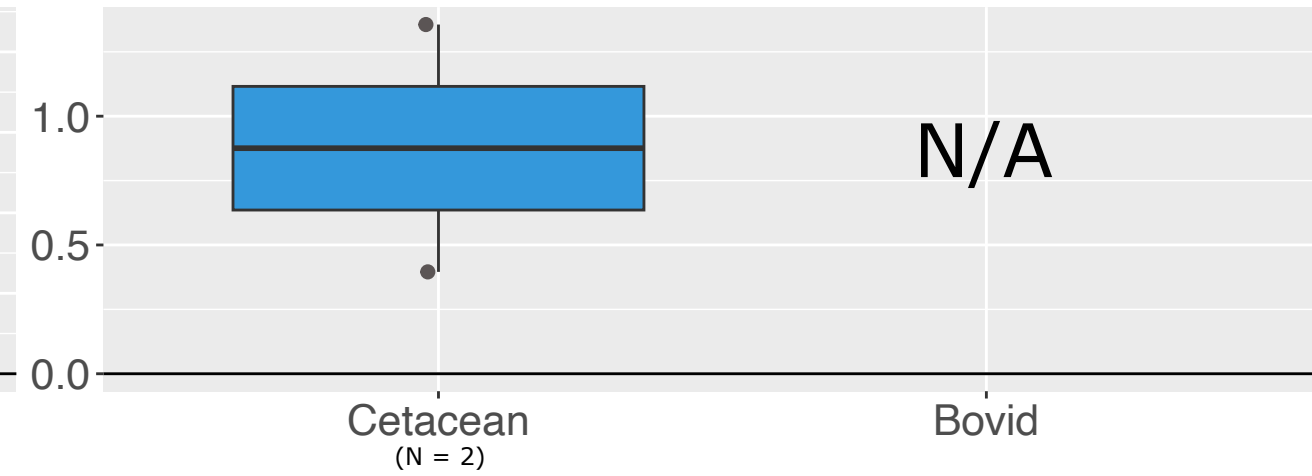

Activity (units/mL)

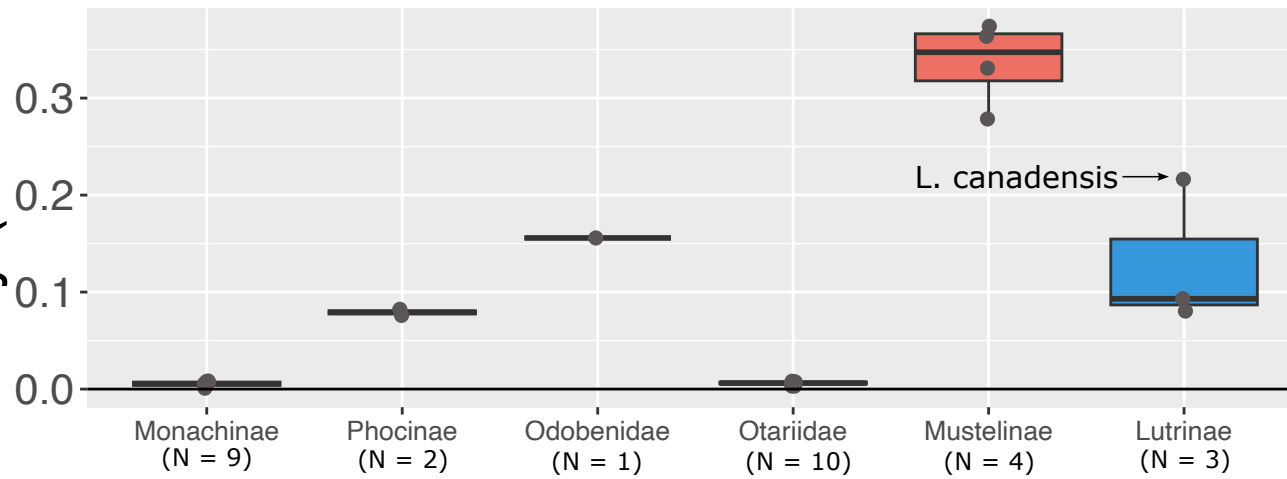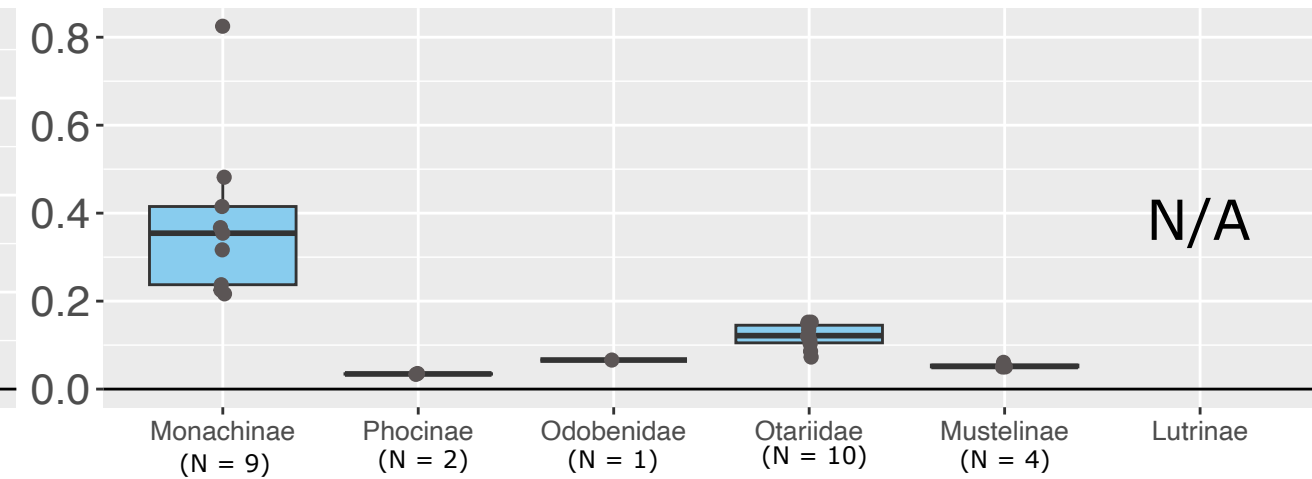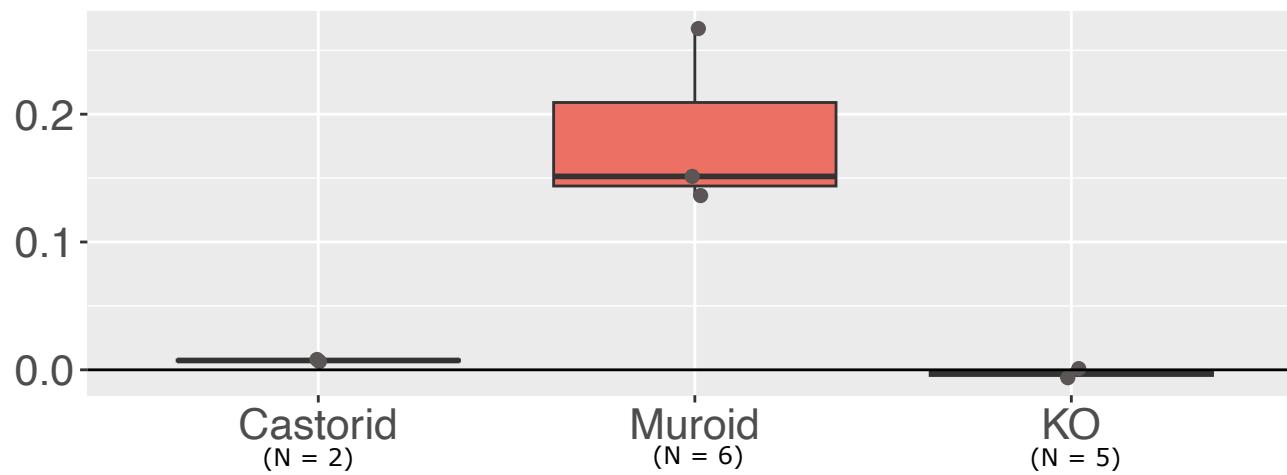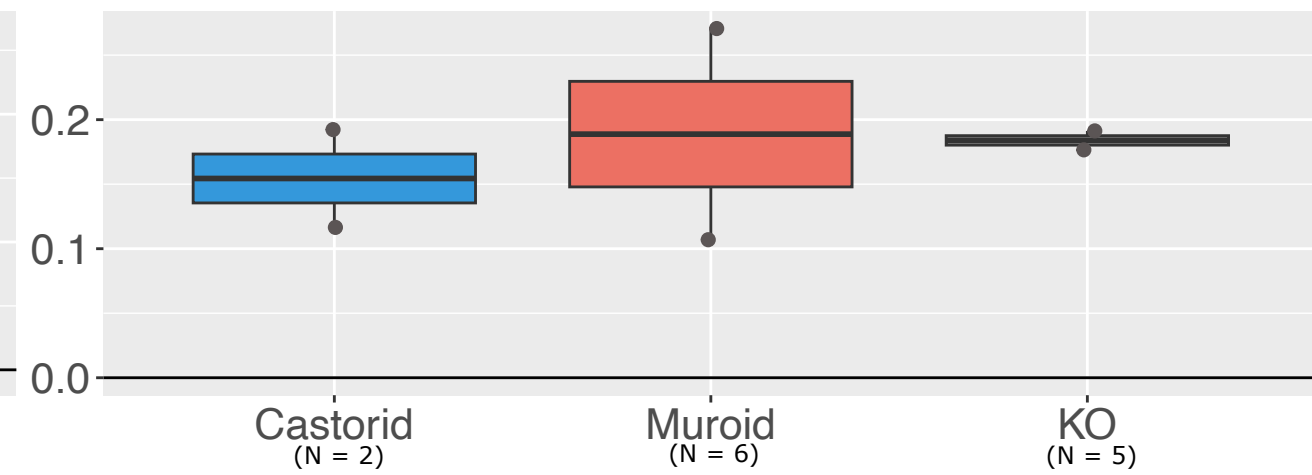

Supplement: msad104_Supplementary_Data [file msad104_supplementary_data.zip › SuppFig1_BoxplotEnzymeActivities_022023_mod.pdf]
